# Cellular senescence, senescence-associated secretory phenotype, and chronic kidney disease

**DOI:** 10.18632/oncotarget.17327

**Published:** 2017-04-21

**Authors:** Wen-Juan Wang, Guang-Yan Cai, Xiang-Mei Chen

**Affiliations:** ^1^ Department of Nephrology, Chinese PLA General Hospital, Chinese PLA Institute of Nephrology, State Key Laboratory of Kidney Diseases, National Clinical Research Center for Kidney Diseases, Beijing 100853, China; ^2^ Department of Nephrology, Beijing Changping Hospital, Beijing 102200, China

**Keywords:** chronic kidney disease, cellular senescence, senescence-associated secretory phenotype, CKD-associated secretory phenotype

## Abstract

Chronic kidney disease (CKD) is increasingly being accepted as a type of renal ageing. The kidney undergoes age-related alterations in both structure and function. To date, a comprehensive analysis of cellular senescence and senescence-associated secretory phenotype (SASP) in CKD is lacking. Hence, this review mainly discusses the relationship between the two phenomena to show the striking similarities between SASP and CKD-associated secretory phenotype (CASP). It has been reported that replicative senescence, stress-induced premature ageing, and epigenetic abnormalities participate in the occurrence and development of CKD. Genomic damage and external environmental stimuli cause increased levels of oxidative stress and a chronic inflammatory state as a result of irreversible cell cycle arrest and low doses of SASP. Similar to SASP, CASP factors activate tissue repair by multiple mechanisms. Once tissue repair fails, the accumulated SASP or CASP species aggravate DNA damage response (DDR) and cause the senescent cells to secrete more SASP factors, accelerating the process of cellular ageing and eventually leading to various ageing-related changes. It is concluded that cellular senescence and SASP participate in the pathological process of CKD, and correspondingly CKD accelerated the progression of cell senescence and the secretion of SASP. These results will facilitate the integration of these mechanisms into the care and management of CKD and other age-related diseases.

## INTRODUCTION

The continuous accumulation of senescent cells leads to the age-related deterioration of vital organs and thus constitutes an organism's ageing process. Correspondingly the therapeutic removal of the senescent cells can improve health and prolong lifespan [1, 2]. Compared with young people, the elderly population not only is more susceptible to kidney damage but also shows more severe clinical manifestations and a lower likelihood of recovery of renal function [3]. Chronic kidney disease (CKD) is increasingly being accepted as a type of renal ageing. Along with the process of ageing, the kidney shows certain types of changes for which specific findings are lacking. The ageing kidney and CKD share a great number of similarities in both structural and functional changes. The structural changes mainly include decreased kidney weight, renal cortical atrophy, increased glomerular sclerosis, tubular atrophy, interstitial fibrosis, capillary loop collapse, glomerular basement membrane thickening, and vascular sclerosis. The functional changes involve a reduced glomerular filtration rate (GFR), increased glomerular capillary pressure, decreased urinary sodium excretion, reduced erythropoietin production, etc [4, 5]. Moreover, progressive degradation in the auto-regulatory capacity and recovery function due to ageing and cellular senescence is closely associated with age-related diseases, especially with CKD.

## CELLULAR SENESCENCE IN CHRONIC KIDNEY DISEASE

CKD is a frequent independent risk factor for renal failure and other age-related diseases. CKD is a complex pathological process mainly involving oxidative stress, inflammation, autophagy, apoptosis, and epigenetics. Recently, cellular senescence has become an increasingly popular and extensively studied topic because of its role in the occurrence and development of CKD. In CKD, such as IgA nephropathy, membranous nephropathy, focal segmental glomerular sclerosis, minimal change disease, diabetic nephropathy, unilateral ureteral obstruction and even in Renal transplantation, the expression levels of senescence-associated β-galactosidase (SA-β-gal) and cell cycle inhibitor p16 protein were significantly increased in the glomeruli, tubules and interstitium, suggesting that the process of cellular senescence occurs in CKD [6–12].

**Figure 1:**
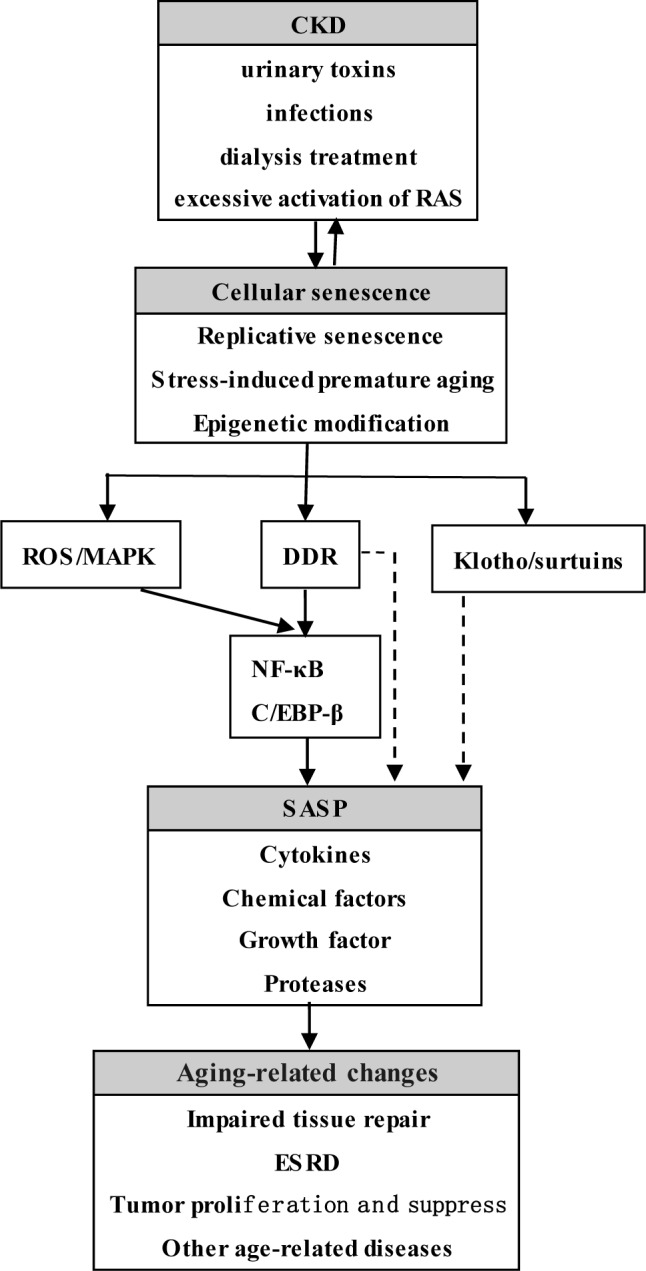
Cellular aging and SASP in CKD Many factors including urinary toxins, infections, dialysis treatment, excessive activation of RAS, can cause and increase telomere shorten, the levels of oxidative stress, and abnormal epigenetic modification, resulting in cell cycle arrest and senescence related morphological changes. Sustained DNA damage response and/or the increased level of ROS directly or indirectly activate transcription factor NF-κB and C/EBP-β and further cause the secretion of SASP. SASP not only can recruit immune cells to clear antigens and the damage cells, but also can launch tissue repair by activating cellular proliferation and differentiation of progenitor cells, stem cells, or the legacy of kidney inherent cell. Once the tissue repair fails, gathered SASP will aggravate DNA damage response and cause the senescent cells to secrete more SASP, accelerating the process of cellular aging and eventually leading to ESRD and other aging-related changes. RAS: Renin-angiotensin system; ROS: Reactive oxygen species; MAPK: Mitogen-activated protein kinase; DDR: DNA damage response; NF-κB: Nuclear transcription factor; C/EBP-β: CCAAT enhancer binding protein β; SASP: Senescence-associated secretory phenotype; ESRD, End-stage renal disease.

Many factors involved in the progress of CKD, such as urinary toxins, infections, dialysis treatment, and excessive activation of the renin-angiotensin system, can cause diverse types of DNA damage response (DDR) and accelerate the ageing process of tubular epithelial cells, immune cells, endothelial cells, progenitor cells, and stem cells [13–17]. The role of cellular senescence in CKD cannot be ignored. Therefore, we will elaborate on the occurrence and development of CKD from the perspective of cellular ageing, the mechanisms of which are shown in Figure 1.

### Replicative senescence

Telomeres shorten with every cell division during replicative senescence, which is involved in renal ageing [18]. In the follow-up of CKD patients with a history of smoking and a history of diabetes, it was found that the CKD risk increased by 44% and 16%, respectively, per 0.1 unit decrease in the telomere length of peripheral blood mononuclear cells [19]. In our laboratory, it has been proven that the leukocyte telomere length shortens with age and is correlated with the glomerular filtration rate and serum levels of cystatin C in various age groups of a healthy population [20]. Further, in CKD patients, a certain correlation is noted between the occurrence and development of CKD and the telomere shortening of peripheral blood mononuclear cells or intrarenal cells. In uraemic predialysis patients, the telomere length of lymphocytes was significantly shorter than that in the control, suggesting the existence of replicative senescence [21]. In uraemic patients undergoing haemodialysis or renal transplantation, the lymphocytes showed shorter telomere lengths and secreted large amounts of inflammatory molecules [18, 21, 22]. Moreover, with the extension of dialysis duration, the telomere length of peripheral blood mononuclear cells gradually shortened, and the expression of p53 correspondingly increased [23].

The telomere length in kidney tissue, especially in proximal tubular cells, has been reported to be correlated with CKD. SA-β-gal staining was significantly increased in the kidneys of CKD cats, while there was no change in their liver or skin [24]. Furthermore, the telomere lengths in proximal tubular epithelial cells and distal tubular epithelial cells, respectively, have been determined. Compared with young cats and geriatric normal cats, proximal tubular epithelial cells in elderly CKD cats have shorter telomere lengths. Additionally, distal tubular epithelial cells in elderly CKD cats have shorter telomere lengths than normal geriatric cats do. No significant telomere shortening in the liver or skin from any group was observed [24]. Therefore, replicative senescence is involved in CKD, but the causal relationship between the two remains under debate.

In summary, it is most likely that the accumulated uraemic toxins cause telomerase activity dysfunction and terminal fusions of chromosomal DNA, resulting in DDR. What's more, DDR exists in senescence cell even without DNA damage. For example, futile hyper-mitogenic drive also lead to DDR during cellular senescence, which is mainly associated with mTOR-dependent mitotic incompetence [25]. Once the DDR is activated, which then leads to cell cycle arrest, redox imbalance, and low-grade chronic inflammation. Cell cycle arrest is not yet senescence and it can follow, or sometimes preced, by geroconversion [26]. Geroconversion is a futile growth depending on growthpromoting, mitogen-/nutrient-sensing pathways such as mTOR [26, 27]. In turn, oxidative stress and chronic inflammation accelerate cellular senescence by interfering with telomerase activity and telomere synthesis.

### Stress-induced premature ageing

In addition to replicative senescence, stress-induced premature ageing is also involved in the progress of CKD. As in replicative senescence, cells undergoing stress-induced premature ageing also exhibit cell hypertrophy, irreversible cell cycle arrest, increased double-stranded DNA breaks, and SA-β-gal staining. Replicative senescence is induced by progressive telomere shortening with every cell division, whereas stress-induced premature ageing is switched on by specific stimuli, including infections, lipopolysaccharides, uraemic toxins, and oxidative stress.

There is convincing evidence that CKD can cause the premature senescence of intrarenal and extrarenal cells, as well as the secretion of various SASP factors, resulting in accelerated cellular senescence and disease progression. Recent studies have reported that endothelial cells play a key role in the premature model of diabetes. Oxidative stress and inflammation are linked to diabetes progression and the development of complications, causing and accelerating the ageing of endothelial cells, which leads to vascular remodelling, including increased vascular stiffness, intimal and medial thickening, and lumen enlargement [7, 14]. Moreover, in diabetic nephropathy models, compared to patients with micro-albuminuria, the senescent cellular phenotype of podocytes and tubular epithelial cells, including the expression of p16 and SA-β-gal staining, was more significant in patients with heavy proteinuria [6, 28, 29]. Correspondingly, *in vitro* experiments have shown that high sugar or glycosylated end products cause ageing of renal tubular epithelial cells and increase their expression of the senescent biomarkers p16 and SA-β-gal [10, 29]. In a unilateral ureteric obstruction mouse model, the expression of erythropoietin clearly decreased, while the expression levels of the senescence marker p16 and SASP factors, such as transforming growth factor-β (TGF-β), in the kidneys significantly increased with the progression of CKD. Recombinant human erythropoietin treatment preserves tubular epithelial cell regeneration and ameliorates renal fibrosis through the dual inhibition of stress-induced senescence and epithelial mesenchymal differentiation [13]. The progressive loss of renal function in predialysis and dialysis patients causes premature ageing of the immune system, especially the dysregulation of circulating T cells [30, 31].

Stress-induced premature ageing is induced by a variety of pathological factors in CKD, such as inflammation, renin-angiotensin-aldosterone system (RAAS) over-activation, uraemia, and haemodialysis. Notably, RAAS plays a central role among the multiple mechanisms involved in renal ageing. The changes in the activity or responsiveness of RAAS that occur with ageing predispose the elderly to fluid and electrolyte imbalances as well as to CKD and acute kidney injury [32]. It has been proven that upon treatment with angiotensin II (Ang II), human glomerular mesangial cells experience premature ageing, presenting with cell cycle arrest, elevated expression of P53 and P21, markedly reduced telomere length, and enhanced SA-β-gal staining, while the Ang II receptor antagonist losartan delays these changes [33]. In addition, Ang II elevates the expression of TGF-β, accelerates the process of epithelial mesenchymal differentiation, and promotes matrix synthesis, eventually leading to glomerulosclerosis and renal fibrosis [33]. Ang II can activate the redox-sensitive transcription factor and nuclear factor-κB (NF-κB) during ageing [34]. However, its specific mechanism remained an open question. Therefore, the relationship between Ang II and NF-κB was further studied, and it was found that NF-κB was activated by increased Ang II through the phosphorylation of nuclear factor of kappa light polypeptide gene enhancer in B-cells inhibitor alpha (IκBα) and p65 [35]. Therefore, systemic chronic inflammation mediated by RAAS disorder can accelerate ageing to some extent. Simultaneously, certain other uraemic factors and related treatments can exacerbate cellular senescence by enhancing chronic, low-grade inflammation and SASP, breaking homeostasis and promoting age-related diseases.

### Epigenetic abnormalities

Epigenetic patterns change over the lifespan, implying that epigenetic abnormalities may constitute a vital component of the aging process [36, 37]. The epigenetic marks that have been widely studies are mainly DNA methylation and modification histones. Abnormalities epigenetic modifications have been proposed as a hypothesis to explain inherited susceptibility to CKD and the increased risk of CKD due to dynamic interactions of the environment with the genome.

DNA methylation is a common type of epigenetic modification in age-related diseases, especially CKD. DNA methylation refers to the combination of a methyl group and cytosine at CpG sites by DNA methyltransferases and is typically associated with gene silencing [38, 39]. It has been reported that uraemic toxin accumulation can silence the anti-ageing protein Klotho through the DNA methyltransferase protein and thus interfere with mineral and vitamin D metabolism, leading to accelerated ageing of the kidney [40]. There is a positive correlation of hypermethylation levels with chronic inflammation as well as with the risk of death in CKD [41]. The hypomethylation of certain genes, especially key renal transcription factors (Hepatocyte nuclear factor, HNF; Transcription factor AP, TCFAP; Sine oculis homeobox homolog 2, SIX2), has been shown to enhance the baseline expression levels of TGF-β, which participates in renal fibrosis [42]. However, in a study by Smyth et al. (2014), pathological hypermethylation was reversed by Tet3-mediated hydroxymethylation, suggesting that endogenous demethylating agents could be transiently activated as a novel therapy for CKD [39]. Therefore, it is speculated that blocking methylation may promote the diagnosis and prediction of individuals at high risk of developing CKD.

The abnormal epigenetic modification of histones also participates in a series of pathological processes involved in CKD, including renal fibrosis, chronic inflammation, hypoxaemia, and certain complications. It has been postulated that the loss of core histones is observed in aged cells and may contribute to ageing [43]. Histone loss accompanies cell division and seems to be initiated by telomeric DNA damage [43]. Sirtuins, the NAD^+^-dependent deacetylase proteases, can be significantly elevated by dietary restriction. In particular, Sirt1, Sirt3 and Sirt6 have been proven to attenuate renal ageing and have been investigated in detail [44, 45]. After blocking the biological activity of Sirt1 by treatment with Tie 2, endothelial cells and progenitor cells appeared to prematurely acquire the senescent phenotype, mainly maintaining vasodilation dysfunction and the inability to form blood vessels [14]. Furthermore, the CKD model was developed based on blocking the activity of Sirt1, and it was observed that the dysfunction of Sirt1 plays an essential role in renal fibrosis [14]. The acetylation of forkhead transcription factor O4 and forkhead transcription factor O1 is decreased by Sirt1 and Sirt3, respectively, and at the same time, the redox balance is improved, delaying the ageing process [46, 47]. The histone modification of NF-κB is regulated by Sirt6, thereby affecting the synthesis and secretion of SASP [48]. However, the relationships among epigenetic modification, cell senescence, and SASP need to be further studied.

## SENESCENCE-ASSOCIATED SECRETORY PHENOTYPE

When cells become senescent, they remain metabolically active and undergo widespread gene expression changes, secreting certain factors and changing the surrounding environment. In 2008, Coppe et al first applied antibody arrays to obtain a quantitative assessment of factors secreted by senescent cells. They were the pioneers who proposed the concept of the senescence-associated secretory phenotype (SASP), consisting of all types of cytokines, chemokines, growth factors, and proteases [49]. Obviously, the specific composition and function of SASP varies according to different environments and different cell types.

When the kidneys suffer injury, the repair mechanisms for the impaired kidney tissue in CKD are started immediately. At present, the cells known to promote tissue repair mainly encompass the residual tubular epithelial cells, mesenchymal stem cells, and/or haematopoietic stem cells. For example, growth factors such as vascular endothelial growth factor (VEGF) and fibroblast growth factor 2 (FGF2), which are significantly increased during kidney diseases, can further promote the proliferation, migration, adhesion, and differentiation of adipose mesenchymal stem cells, endothelial cells, and residual tubular epithelial cells to promote tissue repair [16, 50]. At the same time, the increased expression levels of interleukin- 6 (IL-6), Tumor necrosis factor-α (TNF-α), and monocyte chemotactic protein-1 (MCP-1) recruit a variety of immune cells to promote tissue repair by removing harmful factors, maintaining homeostasis and secreting more SASP [51, 52]. Once tissue repair fails, the accumulated SASP plays various roles in age-related pathological damage.

### Comparison between CKD- and senescence-associated secretory phenotype

In the course of kidney diseases, several cells in the kidney, such as renal tubular epithelial cells, endothelial cells, mesangial cells, podocytes, and immune cells, experience cellular senescence and secrete a large number of factors that are collectively defined as the CKD-associated secretory phenotype (CASP) [7, 15, 16, 30, 53–55]. It has been demonstrated that CASP and SASP have prominent similarities, which may act as an essential medium mediating the interaction between CKD and cellular senescence.

As in the case of SASP and senescence, CASP is not specific to CKD and overlaps with the molecules presented in other kidney diseases or age-related diseases. Therefore, we review the superficial similarities between the CKD-associated secretory phenotype and SASP, as detailed in Table 1 below.

**Table 1:** Comparison between SASP- and CKD-associated secretory phenotype

	Species	SASP[49, 113–116]	CASP
Cytokines and regulators	IL-1, 4, 6, 18	↑*	↑[14, 78, 87, 91, 95, 117]
	TNF-α/TWEAK	↑	↑[91, 95, 118]
	ICAM-1	↑[119]	↑[120, 121]
	VCAM-1	↑	↑[121]
Chemokines	CCL-2,3,5	↑	↑[118, 120, 122]
	IL-8	↑	↑[14, 91, 122]
	GRO	↑	↑[120]
Growth factors and receptors	TGF-β	↑	↑[15, 122, 123]
	VEGF	↑	↑[62, 71, 72, 120, 124]
	MIC-1	↑	↑[14]
	PDGF BB/PDGF receptor-β	↑	↑[123, 125]
	FGF-2, 23	↑	↑[120, 126, 127]
	HB-EGF/EGFR	↑	↑[120, 125]
	CTGF	↑	↑[125]
	GM-CSF	↑	↑[120, 128]
	t-IGFBP3	↑	↑[14, 129]
Proteases	MMP-2,9,7,20	↑	↑[123, 130–132]
	PAI-1	↑	↑[118]
Other factors	iNOS	↑	↑[128]
	ROS	↑	↑[121]
	COX-2	↑	↑[118]
	galectin-3	↑	↑[14]

*Definition of abbreviations*: SASP: senescence-associated secretory phenotype; CASP: Chronic kidney disease-associated secretory phenotype; TNF-α: tumor necrosis factor α; TWEAK: apoptosis of tumor necrosis factor-like weak inducer; ICAM-1: intercellular adhesion molecule 1; VCAM-1: vascular cell adhesion molecule 1; GRO: growth regulators oncogene; TGF-β: transforming growth factor -β; MIC-1: macrophage inhibitory factor 1; VEGF: vascular endothelial growth factor; PDGF: platelet-derived growth factor; FGF: fibroblast growth factor; EGF: endothelial growth factor; GM-CSF: granulocyte-macrophage colony-stimulating factor; IGFBP: insulin-like growth factor binding protein; MMP: matrix metalloproteinase; PAI-1: plasminogen activator inhibitor −1; iNOS: inducible nitric oxide synthase; ROS: reactive oxygen species; COX-2: cyclooxygenase-2.

* Arrows indicate increased levels of secreted factors.

### Production of senescence-associated secretory phenotype

SASP is primarily a property of senescent cells that is caused or accompanied by genomic damage and epigenetic abnormality. Obviously, cellular senescence is initiated by all types of injuries, including ionizing radiation, cytotoxic chemotherapies, topoisomerase inhibitors, oxidative stresses, and other agents [56–58]. Eventually, these stimuli cause single and/or double-strand breaks in DNA, ultimately eliciting DDR and suppressing attempted DNA repair [59–61]. Moreover, certain oncogenes, such as H-RAS, the mitogen-activated protein kinase (MAPK) signalling pathway, and tumour suppressors p53 and retinoblastoma (Rb), are provoked excessively, making DNA damage and cellular senescence inevitable [57, 59, 62]. However, under certain circumstances, DDR can be elicited in the absence of physical DNA damage. For example, histone deacetylase inhibitors activate the DDR protein ataxia-telangiectasia-mutated [63–65]. The DDR, in turn, arrests cell division primarily through activation of the p53/p21 and p16*^INK4a^*/retinoblastoma (Rb) pathways, thereby preventing genomic instability, enforcing senescence growth arrest, and establishing and maintaining SASP [59, 65, 66]. The production of SASP largely depends on the transcription factors NF-κB and CCAAT enhancer binding protein β (C/EBPβ) [65, 66]. Moreover, p38MAPK activation is required for oncogene-induced growth arrest but is independent of DDR. Thus, constitutive p38MAPK activation induces SASP without inducing DDR signalling. Mechanistically, p38MAPK induces SASP at the mRNA level by increasing NF- κB transcriptional activity, whereas it is restricted by p53 [49]. Thus, DDR and NF-κB are essential elements in inducing the production and secretion of SASP.

### Cell specificity and antagonistic pleiotropy

Similar to SASP, CASP can be generated by many cells in CKD. However, it also exhibits cell specificity in different physiological contexts. In CKD, a variety of cells in the kidney, such as renal tubular epithelial cells, endothelial cells, mesangial cells, macrophages, and lymphocytes, can suffer from cellular senescence and secrete large amounts of factors [67–70]. However, the quality and quantity of SASP vary depending on cell type and the different stages.

Under physiological conditions, renal tubular epithelial cells, endothelial cells, and progenitor cells can express VEGF to maintain normal cell functions. Once VEGF is knocked out in mice, the endothelial cells present with hypertrophy, leading to capillary collapse and proteinuria [71]. In CKD patients with proteinuria and decreased GFR, the expression of VEGF is significantly increased mainly in renal tubular epithelial cells. VEGF, as a proinflammatory cytokine, promotes angiogenesis and remodelling, improving hypoxia in the renal tissue but also increasing the risk of renal tumour [71, 72]. Moreover, in the early stages of wound healing, senescent cells and some SASP, such as VEGF, TGF-β, platelet-derived growth factor-AA, and epidermal growth factor, promote wound closure by inducing myofibroblast differentiation and by promoting granulation tissue thickness and collagen deposition [73, 74]. Depletion of p16*^Ink4a^*-positivesenescent cells during wound healing delayed the repair process [1]. In the later stages of wound healing, senescent fibroblasts and TGF-β lead to excessive tissue fibrosis and abnormal epithelial mesenchymal differentiation related with nephropathy [75]. Therefore the evolutionarily selected traits of SASP that ensures fitness early in life can be deleterious at an advanced age, that is the antagonistic pleiotropy theory of ageing [76].

### Roles of diversity

CASP can be induced by different stressors and varying degrees of genomic and epigenomic instability in different cell origins, contributing to their diversity. Consistent with the complexity of the CASP, its biological activities are myriad. With renal ageing, the secreted factors play a key determinant in the attraction, activation, and differentiation of immune cells, active immune surveillance to clear senescent cells, and mobilize stem cells for repair [60, 76, 77] Once tissue repair fails, the accumulated SASP aggravates the DDR and causes the senescent cells to secrete more SASP, accelerating the process of cellular ageing and eventually leading to various ageing-related changes. Inflammatory molecules such as IL-1β and IL-8 are involved in the proliferation of mesangial cells during IgA nephropathy [78]. In the pathological process of focal segmental glomerulosclerosis, the TGF-β pathway is activated in the podocytes and combined with the reactive oxygen species (ROS) in the glomerular endothelial cells, leading to fragmentation of the glomerular capillary loop, massive proteinuria and renal failure, thus indicating that CASP may be the mediator of the crosstalk between podocytes and endothelial cells [79]. Chemokines such as MCP-1 and vascuolar cell adhesion molecule-1 are involved in the recruitment and infiltration of inflammatory cells and promote the proliferation of mesangial cells in the pathogenesis of lupus nephritis [80]. Consistent with angiotensin receptor blockers, growth factors such as FGF23 can significantly reduce the levels of inflammation and oxidative stress and further decrease renal fibrosis and lipid metabolic disorders during the progress of diabetic nephropathy, mainly by blocking the RAAS, enhancing the expression of Klotho, and inhibiting the expression of MCP-1/TNF-α and TGF-β [81, 82]. After the knockdown of the Klotho gene, the expression of MCP-1 significantly increases, and a large number of macrophages and T cells are recruited, which in turn release more inflammatory molecules, resulting in proteinuria and renal function deterioration in the model of salt-sensitive hypertensive disease [83].

SASP has been widely reported to promote on-going chronic, low-grade inflammation that interferes with homeostasis and leads to the development of ageing and age-related diseases, and the same is true of CASP. Both SASP and CASP share the transcription factor NF-κB and show an overlap of molecules, especially IL-1α, IL-6, IL-18, and TNF-α [67, 84, 85]. As a feedback regulator, the inflammatory molecule IL-1α can act on NF-κB, secreting more inflammatory molecules in cascade [86–90]. Compared to healthy controls, the plasma levels of IL-6 and TNF-α rose significantly in CKD stage 2-5 patients, and multiple linear regression analysis showed that plasma TNF-α was negatively correlated with eGFR [91]. The proinflammatory cytokines TNF-α and TNF-like weak inducer of apoptosis, in addition to causing and accelerating inflammatory injury, down-regulate Klotho expression by promoting RelA binding to the Klotho promoter and inducing its deacetylation, resulting in ageing, end stage renal disease, frailty, and other age-related diseases [86, 92]. In addition to age-related degenerative alterations, SASP can also drive hyperplastic pathology. Strikingly, it favours tumour formation and progression through the senescence response, which activates a positive feedback loop and stimulates angiogenesis and fibrosis by recruiting inflammatory immune cells to secrete many proinflammatory and proangiogenic factors [93, 94]. Moreover, a large number of growth factors, cytokines, and certain matrix metalloproteinases (MMPs) also contribute to the process of renal tumour formation by epithelial mesenchymal differentiation, tissue remodelling, and tumour cell invasion [95]. SASP also plays a critical role in the tissue microenvironment in terms of its course of development, wound healing, and renal pathologies.

## AGE-RELATED ALTERATIONS ASSOCIATED WITH CHRONIC KIDNEY DISEASE

Cellular senescence and SASP may be integral mechanisms that mediate CKD and other age-related diseases. Notably, the risk of stroke and unrecognized myocardial infarctions increased linearly and additively with declining GFR and increasing albuminuria during CKD [96, 97]. A study of cardiac magnetic resonance (CMR) imaging revealed that dispensability decreased with increasing age and was reduced at all three thoracic aortic levels in CKD patients, along with decreasing eGFR [98]. The impaired vasodilations in young CKD patients were more severe than that in young healthy controls, whereas microvascular function was similar to that in the elderly population [99]. By stimulating renal tubular epithelial cells with TGF-β, the expression levels of nicotinamide adenine dinucleotide phosphate oxidase and ROS could be significantly up-regulated, indirectly resulting in vascular ageing by reducing the telomere length and activating the p53/21 pathway [19, 23]. There are substantial overlaps between CKD and chronic obstructive pulmonary disease (COPD), such as advanced age, chronic inflammation factors, smoking, and comorbidity with cardiovascular diseases. COPD has been widely regarded as a model of premature senescence. The comorbidity of CKD in COPD patients is approximately 3 times the level in non-COPD patients, suggesting a close correlation between COPD and CKD [100]. A meta-analysis of 63,902 participants shows a significant correlation between the presence and severity of non-alcoholic fatty liver disease and the increased risk and severity of CKD [89]. In addition, DNA damage can lead to an up-regulation of the induction of p21 and p16, which have been shown to limit stem cell function, regeneration, and organ maintenance and to increase cancer risk during ageing. However, the specific mechanisms have yet to be completely identified [101, 102]. Therefore, CKD is tightly related to other age-related diseases through cellular senescence and various other mechanisms. Because CKD is considered to be a multi-factorial disease, there must be other factors besides ageing involved in the apparent reduction in renal function.

## PROSPECTS AND TARGETS OF CELLULAR SENESCENCE AND SASP

Recent studies have shown that both cellular senescence and SASP could be considered as a target to attenuate age-related diseases, mainly by inhibiting the transcriptional activity of NF-κB and reducing the levels of ROS. The most studied drugs, such as glucocorticoids, resveratrol, metformin, rapamycin protease inhibitors, and other drugs, have a wide range of pharmacological effects in the treatment of kidney diseases, especially their anti-inflammation and anti-oxidative stress properties.

### Glucocorticoids

In the model of lupus nephritis caused by lipopolysaccharides, methylprednisolone can reduce the formation of proteinuria, mainly by blocking NF-κB transcription activity and the inhibition of Fractalkine expression [103]. In addition, it has been shown that cortisol and corticosterone could significantly reduce the production and secretion of SASP, such as IL-6, but could not affect the tumour inhibition of SASP. The specific mechanism of action of glucocorticoids is that they block the IL-1α signalling pathway by binding to the glucocorticoid receptor on the cell surface, ultimately inhibiting the transcriptional activity of NF-κB [104].

### Resveratrol

Resveratrol not only reduces oxidative stress by decreasing the levels of ROS, delaying the process of renal fibrosis and reducing glomerular injury and proteinuria in the model of membranous nephropathy and diabetic nephropathy [105, 106], but also significantly decreases the transcription activity of NF-κB and reduces the secretion of IL-1β, IL-6, MCP-1, TNF-α, and other SASP components, ultimately delaying the process of renal ageing [107].

### Metformin

As a drug involved in the regulation of cell metabolism, metformin has been reported to play an increasingly prominent role in delaying cellular senescence. On the one hand, metformin can block the feedback activation of NF-κB by IL-6, TNF-α, and plasminogen activator inhibitor-1. On the other hand, it can also block NF-κB transcription by inhibiting the migration of NF-κB into the nucleus and further blocking the phosphorylation of IκB and IKKα/β. As a result, it ultimately inhibits the cascade amplification of SASP and reduces the occurrence and development of kidney diseases, cardiovascular diseases, and other ageing-related diseases [108].

### Mammalian target of rapamycin (mTOR) inhibitors

Our laboratory has demonstrated that dietary restriction inhibits the expression of mTOR and delays kidney ageing [109]. What’ more, rapamycin inhibits the growth-promoting mTOR pathway and decelerates geroconversion of the arrested cells, thus attenuating aging [26]. Importantly, mTOR inhibitors also selectively suppress the expression of IL-1α on the surfaces of senescent cell membranes, block downstream NF-κB transcription, and reduce the production of SASP, especially IL-6, extending the life of many organisms [110].

### Other

Some traditional Chinese medicines, such as andrographolide, significantly down-regulate the levels of triglycerides, blood glucose, serum creatinine, urea nitrogen, and other factors in the type 2 diabetes model [111]. Andrographolide can also reduce the levels of both oxidative stress and inflammation, delay extracellular matrix deposition, and attenuate glomerular hypertrophy. The mechanism underlying the action of andrographolide is the inhibition of the transcription factor NF-κB [111]. In IgA nephropathy, melatonin can reduce the expression levels of TNF-α, MCP-1, TGF-β, collagen IV, and MMP-9 by inhibiting the transcriptional activity of NF-κB, thus delaying the progress of renal fibrosis and proteinuria [112].

## REMAINING QUESTIONS AND FUTURE ISSUES

Although there is a striking resemblance between SASP and CASP in terms of their features of up-regulation and the species involved, there remain many gaps in the understanding of the complex role of cellular senescence and SASP in CKD and other age-related diseases. It is beneficial to establish their mechanisms in the pathogenesis and progression of CKD. Therefore, the common process of cellular senescence and SASP is considered a treatment target for CKD and other age-related diseases. However, a large number of issues still remain to be discussed, as follows:It has been stressed that the DDR pathway is the common target for the therapeutic modulation of SASP. Therefore, whether a more specific and more sensitive marker for DDR exists to enable the timely blocking or attenuation of the progression of CKD still remains to be known; It is still inconclusive whether CKD or cellular senescence is the causative factor. In particular, there is still uncharted territory between CKD and its complications, such as cardiovascular disease, COPD, tumours, and other age-related alterations; Relatively little is known regarding whether there is a specific accelerated process of cellular senescence, considering that there is rapid deterioration in advanced stages of CKD, and whether this acceleration phase can be used as an opportunity for intervention to attenuate the progression of CKD; More comprehensive knowledge is needed regarding when, where, and how senescent cells, as well as SASP, are beneficial in the processes of tissue repair and regeneration.

## CONCLUSIONS

In general, cellular senescence and SASP participate in CKD and other ageing-related diseases, including degenerative and hyperplastic pathologies. The current means of treating age-related kidney diseases are limited, and there are no specific strategies currently available for elderly patients. Thus, cellular senescence and SASP may provide a target for addressing age-related renal diseases. The specific CASP compositions and mechanisms have not been sufficiently illustrated. Therefore, a comprehensive understanding of cellular senescence and SASP in CKD will provide a new target for the treatment and prevention of CKD.
